# Optical characterization of porcine tissues from various organs in the 650–1100 nm range using time-domain diffuse spectroscopy

**DOI:** 10.1364/BOE.386349

**Published:** 2020-02-28

**Authors:** Sara Mosca, Pranav Lanka, Nick Stone, Sanathana Konugolu Venkata Sekar, Pavel Matousek, Gianluca Valentini, Antonio Pifferi

**Affiliations:** 1 Central Laser Facility, Research Complex at Harwell, STFC Rutherford Appleton Laboratory, UK Research and Innovation, Harwell Campus, OX11 0QX, United Kingdom; 2 Dipartimento di Fisica, Politecnico di Milano, Milano, Italy; 3 School of Physics and Astronomy, University of Exeter, Exeter, EX4 4QL, United Kingdom; 4 Consiglio Nazionale delle Ricerche, Istituto di Fotonica e Nanotecnologie, Milano, Italy; 5 Biophotonics@Tyndall, IPIC, Tyndall National Institute, Lee Maltings, Dyke Parade, Cork, Ireland; 6 These authors contributed equally to this research

## Abstract

We present a systematic characterization of the optical properties (µ_a_ and µ_s_’) of nine representative ex vivo porcine tissues over a broadband spectrum (650-1100 nm). We applied time-resolved diffuse optical spectroscopy measurements for recovering the optical properties of porcine tissues depicting a realistic representation of the tissue heterogeneity and morphology likely to be found in different ex vivo tissues. The results demonstrate a large spectral and inter-tissue variation of optical properties. The data can be exploited for planning or simulating ex vivo experiments with various biophotonics techniques, or even to construct artificial structures mimicking specific pathologies exploiting the wide assortment in optical properties.

## 1. Introduction

The knowledge of optical properties of biological tissues is vital in biomedical optics research, as it underpins the design of effective devices and methods [1,2] or planning therapeutic protocols [3,4] and it is also crucial for interpreting diagnostic measurements [5]. The optimization and characterization of biophotonics systems are often carried out using tissue-mimicking optical phantoms [6–8]. Synthetic phantoms can be made both in solid or liquid forms with the aim of mimicking the optical properties of the main tissue component (i.e. water, lipid) over the spectral range of interest [6,9]. These are easy to handle in routine instrument validation with the advantage of stable and reproducible optical properties, however, they mimic rather poorly mechanical properties and real and complex heterogeneities present in human organs. In contrast, biological phantoms, made from animal tissues, demonstrate better the degree of heterogeneity and morphological complexity common in biological tissues [10]. In addition, they have a set of major chromophores typically present also in human tissues that are not easily modelled with synthetic phantoms. For the reasons mentioned above, porcine tissues can serve as versatile biological phantoms as they share similar anatomic and physiologic characteristics with humans, but are easily accessible with respect to ex vivo human tissues and present a lower level of hazard. Also, heterogeneous structures (e.g. a localized inhomogeneity mimicking a tumor within an organ) can be constructed by combining different tissue types. The in-depth knowledge of the optical properties of biological phantoms can be used as a starting point for creating accurate models of light propagation or to test the capability of the methodology/system for retrieving the optical properties [11,12].

In recent years, the applications of different biophotonics techniques in medicine as non-invasive diagnostic tools [13–15] and light-guided therapy [3,16] have increased dramatically. Within this context, the area of biological tissue phantoms has a direct impact on several research fields such as near-infrared spectroscopy and tomography [17–19], photoacoustic imaging [20,21], diffuse Raman spectroscopy [22–26], fluorescence spectroscopy [27–30] and photodynamic therapy [31,32]. Unfortunately, sparse and often inconsistent values of optical properties are available for tissues of different animal origin (i.e. rat, canine, sheep, horse, porcine, bovine, chicken, human) which are often only reported at sporadic wavelengths [10,33,34]. Moreover, these results are obtained with different experimental and theoretical approaches [35,36] using continuous-wave [10,33,37], time-domain [38–40] or frequency-domain [41–43] systems, often leading to inconsistent data sets.

Here we perform the optical characterization of a large number of fresh porcine tissues over a wide spectral range within the visible and near-infrared region (i.e. 650 nm - 1100 nm). We use Time-domain Diffuse Optical Spectroscopy (TD-DOS) which enables unambiguous dis-entanglement of absorption and reduced scattering coefficients of tissues [11] and can also be implemented over a broad spectral range in the near-infrared spectral region [18]. Furthermore, since the retrieval of optical properties for a homogeneous sample relies only on the temporal shape independently of the signal amplitude, this technique is not affected by uncertainties in calibration or optical contact. This work represents the first systematic investigation of fresh ex vivo tissue taken from the animal type across such a range of parameters. The selection of tissues is not exhaustive but sufficiently representative including dermal (e.g. skin), nervous (e.g. brain), connective (bone, tendon, adipose), muscular (e.g. muscle) and other organs (e.g. kidney, heart. lung).

Surely, the ex vivo tissues are altered as compared to the in vivo environment [44]. In particular, one can expect exsanguination with reduction of total blood, oxidative changes in hemoglobin and an increase in scattering when shifting to room temperature. Also, in the case of freezing-thawing cycles, cell membrane disruption will lead to scattering alterations and liquid loss. As far as possible, we tried to avoid big alterations, but definitely, the ex vivo tissue cannot be taken as a perfect replicate of the in vivo situation, also because of the differences between the porcine organs and the human case. Yet, the ex vivo tissues are still a better approximation to the in vivo case with respect to synthetic phantoms, and the collections of the tissues presented below can be considered as a kind of library for picking the best tissues to mimic the in vivo scenario.

## 2. Materials and methods

### 2.1 Instrumentation

**Fig. 1.**
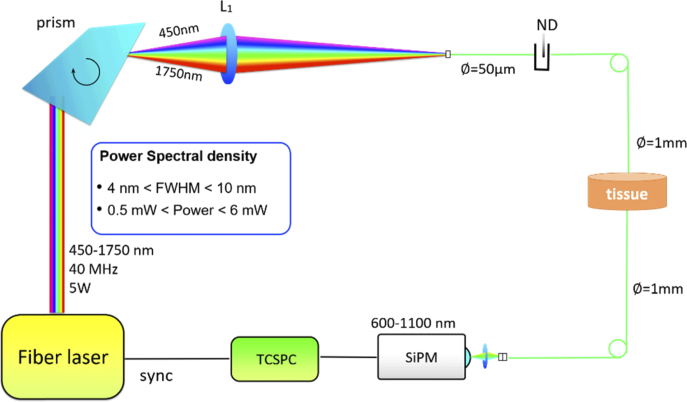
Schematic of the of time resolved diffuse optical instrumentation used in this study.

The absorption (µ_a_) and reduced scattering coefficient (µ_s_’) were measured using a broadband time-domain diffuse optical Spectroscopy (TD-DOS) system built by Politecnico di Milano [45]. Figure 1

shows the schematic of the experimental setup. A detailed description of the instrumentation is presented elsewhere [18,45]. A fiber-based supercontinuum laser (SuperK EXTREME (EXW-12), NKT Photonics, Denmark) was used as the illumination source. Broadband picosecond pulses (450–1750 nm, repetition rate = 40 MHz) from this source were collimated and impinged on a dispersive Pellin-Broca prism that achieved wavelength selection through the rotation. The linewidth of the output varied from 3nm at 650 nm to 9 nm at 1100 nm. This wavelength-selected light was then focused into a 50 µm graded-index fiber. A set of neutral density circular attenuators were used to control the light power. Thus, after sufficient attenuation, the light was coupled to a 1 mm step-index fiber placed in gentle contact with the sample. Light diffusively transmitted from the sample was then collected using another 1 mm step-index fiber and was focused into a custom made Silicon PhotoMultiplier (SiPM) module with a good photon harvesting capability over the wavelength range of interest (650–1100 nm) [46,47]. The FWHM of this detector’s Instrument Response Function was under 100 ps over the entire wavelength range. The signal acquired was processed using a time correlated single photon counting (TCSPC) board, (SPC-130, Becker & Hickl, Germany), and the produced temporal point spread functions (TPSFs) were saved in a PC. The entire system was automated for faster acquisition and controlled using in-house software. The system has been characterized, validated using internationally agreed protocols [48,49] and employed across various phantom [9,24] and clinical studies [50].

### 2.2 Samples

**Fig. 2.**
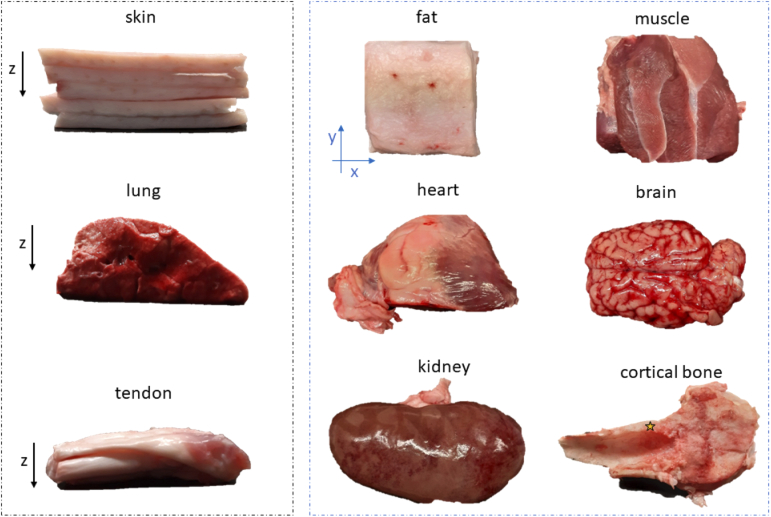
Photos of all 9 porcine ex-vivo samples. The yellow star in the cortical bone panel indicates the analyzed point.

The pictures of the analyzed ex vivo porcine samples are presented in Fig. 2

. All the tissue phantoms were obtained from the same animal type. Samples were refrigerated during storage (approx. 4 °C) and allowed to return to room temperature (21 °C) before the measurements. The optical characterization was repeated for all the tissues in three days, within six days of sacrificing the animal. The samples were sandwiched between two custom made black PVC panels that held them in position. Each plate had a small opening to host the source and detector fiber and maintain them at a position just in contact with the sample. Care was taken to ensure that the PVC panels did not squeeze the sample thereby changing its structural and optical properties. The measurements were performed on a portion or on the whole organ tissues in correspondence of a thickness of around 20 mm (the exact sample dimensions are reported as *z* in Table 1

**Table 1.** List of porcine samples (organ, dimensions, comment if any).

Samples	Geometrical dimensions (cm)			Comment
	z	x	y	
Skin	2.2	5.5	6.0	Overlap of 5 layers
Fat	2.0	6.3	6.5	Overlap of 2 layers
Muscle	2.0	7.5	6.5	Portion
Lungs	2.0	7.5	6.0	Portion
Heart	2.0	4.5	5.5	Portion
Brain	2.0	9.8	6.0	Whole organ
Tendon	1.0	6.1	2.1	Overlap of 6 layers
Kidney	2.5	14.0	8.5	Whole organ
Bone*	0.8*	4.5	2.7	*Refers to a cortical bone segment

). The values of the thickness were chosen, after preliminary measurement, as a good compromise between the signal level over the entire spectrum and the validity of the diffusion equation [51]. Also, the lateral dimensions (x and y in Table 1) were chosen to be as large as possible to avoid boundary effects.

### 2.3 Measurement protocol and data analysis

The measurements of the ex vivo porcine tissues were performed in transmission geometry along the z dimension of the sample (Table 1). As mentioned earlier, the value of the sample thickness was around 20 mm in most cases, to allow for proper diffusion of the light through the sample. The temporal photon distributions were acquired in the spectral range from 650 nm to 1100 nm at steps of 10 nm. Measurements were performed on three different locations of each tissue to account for intra-sample spatial variation of the optical properties. Acquisition time per wavelength was 4 seconds and about 5 minutes for an entire spectrum.

The acquired TPSFs at each wavelength were fit to an analytical solution of the Radiative Transport Equation under the Diffusion Approximation with extrapolated boundary conditions [51] to retrieve the optical properties of the tissue. The sample was considered to be a homogeneous infinite slab with a finite thickness (z coordinate). The refractive index of the sample was assumed to be 1.44 (average value for biological tissue) with an external index of 1.53 to account for the PVC plates with the exception of bone, tendon and skin where an air interface is more appropriate due to the irregular surface. The Instrument Response Function (IRF) was acquired once every hour during the experiment to account for any broadening due to the instrument’s performance and to infer the time origin *t_0_* of the analytical solution used to fit the data. The theoretical model was convolved with the IRF and fitted to the measured data using a nonlinear Levenberg-Marquardt optimization algorithm to estimate the absorption and reduced scattering coefficients. The fitted range in the TPSF covered from above 80% of the peak value on the rising edge, down to 1% on the trailing edge. The chi-square residual of the fit was considered as the figure of merit to decide the accuracy of the retrieved optical properties [52]. The processing time for each retrieval (one point in the spectrum) was under 1 second.

## 3. Results and discussion

**Fig. 3.**
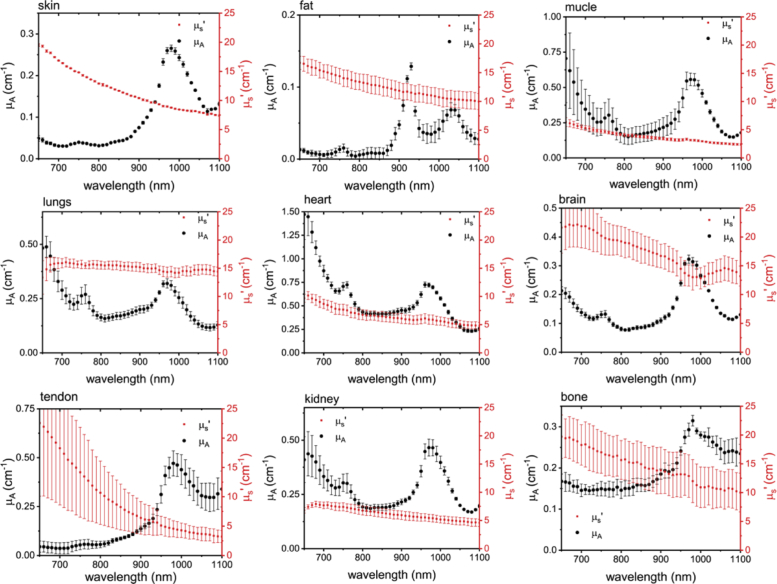
Absorption (black dots) and reduced scattering spectrum (red squares) of the 9 porcine samples. The values are averages of three repetitions; y-error bars display the standard deviation. See **Data Files Mua.txt and Musp.txt in** Dataset 1 [55] and Dataset 2 [56], respectively for underlying values.

The absorption (black dots) and reduced scattering (red squares) spectra of the nine types of tissue considered for this study are presented in Fig. 3

. The values reported in the spectra embody the mean of the three spatially separated measurements and the error bars represent the standard deviation. The results show a robust reconstruction of absorption/reduced scattering coefficients within all the repetitions for all the samples (no significant trend among the three repetitions was observed).

The absorption coefficient spectra have two consistent spectral peaks over most of the tissue types considered (except for the fat tissue). These are, i) a clearly distinguishable peak at 980 nm and ii) a relatively smaller and subtler peak at 760 nm. Both peaks have been well characterized and have been attributed to the water and blood content of the tissue, respectively. Other specific features are the double peak at 930 nm and 1040 nm for the fat tissue, representative of the lipid content [53] and the relatively broader peak around 980 nm for the bone which could be due to a combination of water and high amount of collagen [19] present in this type of tissue. Also, the high absorption observed in some of the tissue types close to the red spectral range (around 650 nm) can be a signature of higher blood content in these tissues.

Figure 3 allows for a comparison of the optical properties amongst the different tissue types. Here, the tissues can be classified into two categories based on the average absorption coefficient values under 900 nm. Skin, fat, tendon, brain and bone are the less absorbing tissue types with an average absorption value under 0.2 cm^−1^ in this region. Lung, kidney, muscle and heart tissue display an absorption higher than 0.2 cm^−1^ on average with heart tissue being the highest. This is reasonable given that all these tissue types are comprised of large amounts of water and blood which are the key absorbers in the therapeutic wavelength window.

Reduced scattering coefficient spectra, in general, display a smoothly decreasing trend with wavelength for all the tissue types. We remind the reader that the reduced scattering coefficient $\mu_{\text{s}}^{\prime }$ is related to the scattering coefficient $\mu_{\text{s}}$ by the formula $\mu_{\text{s}}^{\prime }=\mu_{\text{s}}(1-\text{g})$ where *g* is the mean cosine of the scattering angles. Therefore, $\mu_{\text{s}}^{\prime }$ represents an effective scattering coefficient assuming perfectly isotropic interaction (g=0). This behavior has been studied extensively [54] and is approximated by an empirical power-law derived from the Mie theory of the form $\mu_{\text{s}}^{\prime }(\lambda )=a(\lambda /\lambda_{\text{o}}{)}^{-\text{b}}$, where a and b are the scatter amplitude and scatter power which are related to the density and size of the scattering centers. The slope of the reduced scattering spectra shows a marked variation amongst the different tissue types (red dots in Fig. 3). Tendon and skin tissues show a steep decrease in the reduced scattering coefficient with wavelength compared to the other tissue types. On the contrary, the lung tissue shows the least change in the reduced scattering coefficient with increasing wavelength. There is also sufficient dispersion in the magnitude of the reduced scattering coefficient spectrum. The brain tissue owing to its unique structure presents a relatively higher magnitude of reduced scattering ranging between 20 to 15 cm^−1^ while the muscle tissue has a reduced scattering coefficient under 5 cm^−1^ over the entire range. This huge variation, both in the magnitude and shape of the reduced scattering coefficient spectra, among different tissues could be due to the difference in the shape and structure of the scattering centers of the different tissues considered.

**Fig. 4.**
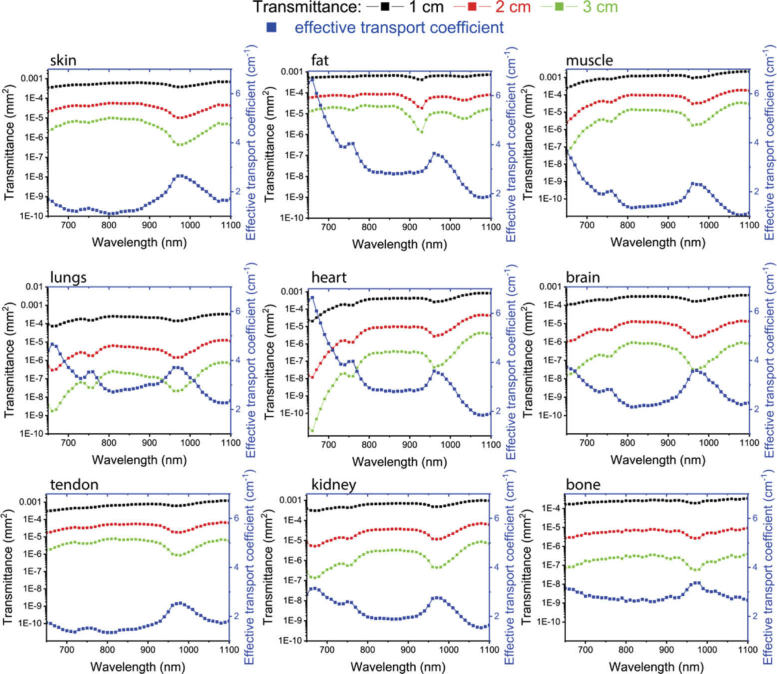
Calculation of the transmittance (left y-axes) of CW light for 1 cm (black filled square), 2 cm (red filled square) and 3 cm (green filled square) thick slab of the different tissues studied. Effective transport coefficient (blue filled square, right y-axes).

To compare the signal attenuation produced by different tissue types, Fig. 4

displays the effective transport coefficient $\mu_{\text{eff}}=\sqrt{3\mu_{a}\mu_{s}^{\prime }}$ (right axis) and the transmittance (left axis) calculated through slabs of 3 different thicknesses for the 9 samples [57]. All values were derived using the data presented above, and using the solution of the diffusion equation under extrapolated boundary conditions [51]. More specifically, $\mu_{\text{eff}}$ is the key parameter governing propagation when considering only continuous wave (CW) signals – which are affected by the combined effects of both $\mu_{a}$ and $\mu_{s}^{\prime }$. The spectral features of $\mu_{\text{eff}}$ shown in Fig. 4 resemble those of $\mu_{a}$, while the overall magnitude and slope is affected by $\mu_{s}^{\prime }$.

Conversely, the transmittance value can be interpreted as the fraction of injected photons (assuming a point source) exiting on the opposite side of the slab for a unitary surface of 1 mm^2^. Referring again to Fig. 4, there is a huge orders-of-magnitude difference in signal attenuation related to the tissue type, wavelength and thickness. Data presented in Fig. 4 can be used as a first-approximation tool to guess the signal attenuation in practical cases and help in the design of experiments. These results hold only for the transmittance case, while to derive information on other parameters and geometries (e.g. fluence rate at a given depth within a semi-infinite medium) specific models must be used based on $\mu_{\text{eff}}$ or directly on $\mu_{a}$ and $\mu_{s}^{\prime }$.

## 4. Conclusion

We have presented a robust, systematic and broadband (650-1100 nm) characterisation of porcine ex vivo tissues and organs using TD-DOS. The results obtained are consistent with the sporadic data available in previous works [10,34] in terms of wavelength dependence and order of magnitude of tissues optical properties. Absorption coefficient below 900 nm of heart, kidney, muscle and lungs was found to be higher than 0.2 cm^−1^ due to the high presence of blood. Large degree of dispersion of reduced scattering coefficient values was measured among different ex vivo tissues. Particularly, tendon shows the steepest decrees of the reduced scattering in the 650 nm – 1100 nm spectral region going from 30 cm to 5 cm^−1^.

The results presented here can be used for creating accurate light transport models for predicting the light propagation and for the optimisation of experimental parameters in biophotonics in general. Having access to the optical properties (µ_eff_, µ_a_ and µ_s_’.) of the same animal species allows one to create a model that mimics heterogeneous structure by combining different tissue types. Moreover, the spectral information within the near-infrared region (650-1100 nm) allows one to optimize experimental parameters (e.g. excitation wavelength) for a specific purpose. For example, they can guide the selection of the excitation wavelength for light-guided therapy in a specific organ or they can help to model both the illumination wavelength and the shifted output of Raman spectroscopy measurements on a lesion buried in-depth within biological tissues. The presented spectra are available for downloading in electronic format within the supplementary material Dataset 1 [55] and Dataset 2 [56].
